# Long-Term Mortality in Patients with Tuberculous Meningitis: A Danish Nationwide Cohort Study

**DOI:** 10.1371/journal.pone.0027900

**Published:** 2011-11-22

**Authors:** Anne-Sophie Halkjær Christensen, Casper Roed, Lars Haukali Omland, Peter Henrik Andersen, Niels Obel, Åse Bengaard Andersen

**Affiliations:** 1 Department of Infectious Diseases, Copenhagen University Hospital, Rigshospitalet, Copenhagen, Denmark; 2 Department of Epidemiology, Statens Serum Institut, Copenhagen, Denmark; 3 Department of Infectious Diseases, Odense University Hospital, Odense, Denmark; McGill University, Canada

## Abstract

**Background:**

With high short-term mortality and substantial excess morbidity among survivors, tuberculous meningitis (TBM) is the most severe manifestation of extra-pulmonary tuberculosis (TB). The objective of this study was to assess the long-term mortality and causes of death in a TBM patient population compared to the background population.

**Methods:**

A nationwide cohort study was conducted enrolling patients notified with TBM in Denmark from 1972–2008 and alive one year after TBM diagnosis. Data was extracted from national registries. From the background population we identified a control cohort of individuals matched on gender and date of birth. Kaplan-Meier survival curves and Cox regression analysis were used to estimate mortality rate ratios (MRR) and analyse causes of death.

**Findings:**

A total of 55 TBM patients and 550 individuals from the background population were included in the study. Eighteen patients (32.7%) and 107 population controls (19.5%) died during the observation period. The overall MRR was 1.79 (95%CI: 1.09–2.95) for TBM patients compared to the population control cohort. TBM patients in the age group 31–60 years at time of diagnosis had the highest relative risk of death (MRR 2.68; 95%CI 1.34–5.34). The TBM patients had a higher risk of death due to infectious disease, but not from other causes of death.

**Conclusion:**

Adult TBM patients have an almost two-fold increased long-term mortality and the excess mortality stems from infectious disease related causes of death.

## Introduction

Tuberculous meningitis (TBM) is the most devastating manifestation of extra-pulmonary tuberculosis (TB) being fatal if left untreated. Even with standard anti-tuberculous therapy short-term mortality is high; ranging from 20–69% [1]–[4]. We have recently reported a 19% short-term mortality and a high risk of sequelae (50%) in TBM patients in Denmark over the last decade [4].

The acute, short-term mortality rates in TBM are well documented, but few studies have analysed long-term mortality. In this population based cohort study the long-term mortality in TBM patients was compared to a population control cohort in a TB and human immunodeficiency virus (HIV) low-endemic country with equal access to free, public health care.

## Materials and Methods

### Ethics statement

This study is a registry based cohort study not involving animals or human subjects – therefore an approval from the Ethical Committee has not been applicable. The usage of data from the various registries has been approved by the Danish Data Protection Agency.

### Study design

This study was conducted as a population based nationwide cohort study. The study populations were all TBM patients registered in Denmark in the period 1972–2008 and a cohort of gender and age matched population controls. Outcome was time to death and to cause-specific death.

### Setting

As of January 2008 Denmark had an estimated population of 5.5 million [5]. The reported incidence of tuberculosis (TB), a mandatory notifiable infectious disease in Denmark, was 6/100,000 in 2009 with 329 new notified cases out of which 65% occurred in immigrants [6]. The incidence of TB in Denmark declined from the early 1970's (14/100,000) to the middle of the 1980's (4/100,000) where the incidence rose due to an increase in immigration from TB-endemic countries [7]. The incidence peaked in 2000 at 10.3/100.000 with 548 new notified cases [8]. The notified number of TBM cases has consistently been five to six per year over the last three decades. Denmark has an estimated HIV prevalence of 0.07% [9]. Throughout the study period all Danish citizens had free and equal access to public sector health care and all prescribed anti-tuberculous medications were available free of charge. The Bacille Calmette Guerin (BCG) vaccine usage was phased out of the Danish national childhood vaccination program in the period 1976–1980.

### Data sources

The unique 10-digit Central Person Registration (CPR) number assigned to all Danish citizens at birth or immigration was used to avoid multiple registrations and to track individuals in the following registries.

### The Danish Tuberculosis Registry (DTBR)

All TB patients are notified to Statens Serum Institute, Department of Epidemiology, by the treating physician. This notification is mandatory for all patients initiating anti-tuberculous treatment.

Data from the DTBR is available from two separate registries; a historical registry covering all notified cases from 1972–1987 and the present registry initiated in 1990. Due to a transition period between the two registries data from the calendar years 1988 and 1989 are missing.

The DTBR contains data on the patient's CPR number, age, anatomical localisation of TB, ethnic origin, date of diagnosis, residence, and, if known, source of TB exposure and/or information on relapse as well as microbiological data.

In Denmark all TB diagnostics, including microscopy, nucleic acid amplification (NAA), culture, genotypic identification and mutation analyses, are centralised at Statens Serum Institute's International Reference Laboratory of Mycobacteriology.

### The Danish National Patient Registry (DNPR)

DNPR contains information on all patients discharged from Danish somatic hospitals since 1977 [10]. In 1995 the registry was expanded to also include psychiatric admissions. Data available includes the patient's CPR number, hospital and ward, dates of admission, and discharge diagnosis codes. These codes follow the *International Classification of Diseases*, Eighth Revision (ICD-8), until the end of 1993 and the Tenth Revision (ICD-10) thereafter. The physician can code one primary and up to 19 secondary discharge diagnoses. From this registry, date of first admission with TBM was extracted.

### Civil Registration System (CRS)

The CRS was established in 1968 and contains information on vital status, residency, immigration and/or emigration status on all Danish residents [11]. The following data was extracted: date of birth, gender, date of immigration or emigration, location of residence and date of death.

### Danish Registry of Causes of Death (DRCD)

DRCD contains data from all Danish death certificates since 1943 [12] – the registry is currently updated to 31 December 2009. Causes of death are coded according to the ICD-8 until the end of 1993 and according to the ICD-10 thereafter. The physician in charge can code one immediate cause of death followed by secondary and/or tertiary causes of death and finally one underlying cause of death. The underlying cause of death was in our study considered to be the specific cause of death and these codes were extracted for all TBM patients and population controls that died and categorised as listed in Appendix S1.

### Study population

#### Tuberculous meningitis patients

We included all patients who: 1) were notified to the DTBR from 1 January 1972 until 31 December 2008 and 2) were registered in the DNPR with a diagnosis of TBM or tuberculoma (ICD-8 codes 013.00–014.00 and the following ICD-10 codes: A 17.0–17.1, A 17.8A, A 17.8D) and 3) were ≥16 years of age at TBM diagnosis, and 4) were registered in the CRS. The date of TBM diagnosis was defined as the date of first admission for TBM according to DNPR, and the index date as one year after TBM diagnosis. Patients were excluded from the study population when they died, emigrated or were lost to follow-up within the first year after TBM diagnosis.

#### Population control cohort

From the CRS we identified ten random population controls for each TBM patient included in the study population. Each population control was matched on gender and date of birth (month and year) with the corresponding TBM patient; also, the population controls had to be alive and living in Denmark on the index date (i.e. one year after diagnosis) of the respective TBM patient. The index date for the population controls was hence the same as for the TBM patient to whom they were matched.

### Comorbidity

A modified Charlson Comorbidity Index (CCI) score was used as a measurement of comorbidity. This score is derived from DNPR diagnoses prior to the first TBM diagnosis. A score of 1, 2, 3 or 6, is assigned to a range of co-morbid conditions, such as ischemic heart disease, cancer, liver disease or acquired immune deficiency syndrome (AIDS). This score is correlated to the mortality associated with the condition [13]. The sum of individual scores serves as a comorbidity parameter. Three levels of comorbidity were defined: none (CCI score = 0), low (CCI score = 1–2) or high (CCI score>3).

### Outcome

Primary study outcome was time from index date to death and cause-specific death.

### Statistical analysis

We used chi-square or, where appropriate, Fischer's exact test to compare categorical data for cases and controls. Continuous data was compared using the Kruskal-Wallis test. Time was calculated from index date to the date of death, emigration, loss to follow-up or 31 December 2009, whichever came first. Kaplan-Meier analyses were used to construct survival curves. Cox proportional hazard regression analyses were used to calculate mortality rate ratios (MRR). Schoenfeld plots confirmed that the proportional hazard assumptions were fulfilled. MRR were adjusted for Charlson Comorbidity Index score (categorical ordinal variable) as calculated up to two years prior to TBM diagnosis to minimise risk of confounding. SPSS version 15.0 for Windows and R version 2.13 were used for data analysis. The study was approved by the Danish Data Protection Agency.

## Results

### Characteristics of the study population

In the period 1972–2008 a total of 127 patients were identified with TBM in DNPR and DTBR. Within the first year of diagnosis, 45 patients died (32%), two emigrated and 25 were less than 16 years of age at diagnosis leaving 55 in the study population.

Baseline characteristics of the 55 patients and their 550 population controls are shown in Table 1. A total of 42 cases (76.4%) had a microbiologically verified diagnosis (either through microscopy, NAA or culture). There was an equal sex distribution among the TBM patients and the median age at time of TBM diagnosis was 39.5 years (IQR: 29.0–56.4 years). More than half the patients were between 31 and 60 years of age at time of diagnosis. Thirty percent of the immigrant patients were between 16–30 years at time of diagnosis whereas only 20% of the Danish patients were in this age category. Likewise, we found a higher percentage of Danish patients (28%) versus immigrants (10%) in the age category >60 years at time of diagnosis.

**Table 1:** Characteristics of tuberculous meningitis (TBM) patients and population controls, Denmark 1972–2008.

	TBM patients	Controls	P-value
Total included, no	55	550	
Males, no (%)	27 (49.1)	270 (49.1)	
Ethnic Danes, no (%)	25 (45.5)	492 (89.5)	<0,001
Observation time (total), yrs	668.5	7242.3	
Observation time, median (IQR), yrs	8.8 (4.39–19.64)	10.46 (5.68–22.18)	
Lost to follow-up during study period, no (%)	1 (1.8)	0	
Emigrated during study period, no (%)	2 (3.6)	20 (3.6)	
Age at diagnosis, median (IQR), yrs	39.5 (29.0–56.4)	39.5 (29.0–56.4)	
Age at diagnosis by age intervals, no (%)			
16–30 yrs	14 (25.5)		
31–60 yrs	31 (56.4)		
>61 yrs	10 (18.2)		
Patients diagnosed by decade, no (%)			
1972–1980	7 (12.7)		
1981–1990	15 (27.3)		
1991–2000	17 (30.9)		
2001–2010	16 (29.1)		
Age at diagnosis by decade, median (IQR), yrs			
1970–1980	42.8 (28.5–67.1)		
1981–1990	36.9 (25.1–54.8)		
1991–2000	43.7 (32.9–56.4)		
2001–2011	39.6 (30.9–53.7)		
Modified Charlson Comorbidity Index 2 years prior to TBM diagnosis, no (%)			
None (score = 0)	52 (94.5)	524 (95.3)	
Low (score = 1–2)	2 (3.6)	25 (4.5)	
High (score≥3)	1 (1.8)	1 (0.2)	
Tuberculosis diagnosis prior to TBM	15 (27.3)	1 (0.2)	<0,001
HIV diagnosis prior to TBM diagnosis	4 (7.3)	0	

There were fewer patients diagnosed in the 1970's compared to the three other decades. The age at time of diagnosis was, however, evenly distributed in the four decades. The level of comorbidity two years prior to TBM diagnosis did not differ between the TBM and the control population; a total of 5,4% of the TBM patients had either a low or high Modified Charlson Comorbidity Index compared to 4,7% in the population control cohort. Four of the TBM patients (7.3%) were HIV positive compared to none in the control group.

The number of TBM patients with a known TB diagnosis prior to time of TBM diagnosis was 15 (27.3%) – compared with only 1 (0.18%) case in the population control cohort. Nine (60%) of these patients with a history of TB were immigrants and ten (66.7%) had been diagnosed with TB within one year prior to TBM. The specific previous TB diagnoses were pulmonary TB (7 cases, 46.7%) and extra-pulmonary TB (8 cases, 53.3%).

### Overall mortality

**Figure 1:**
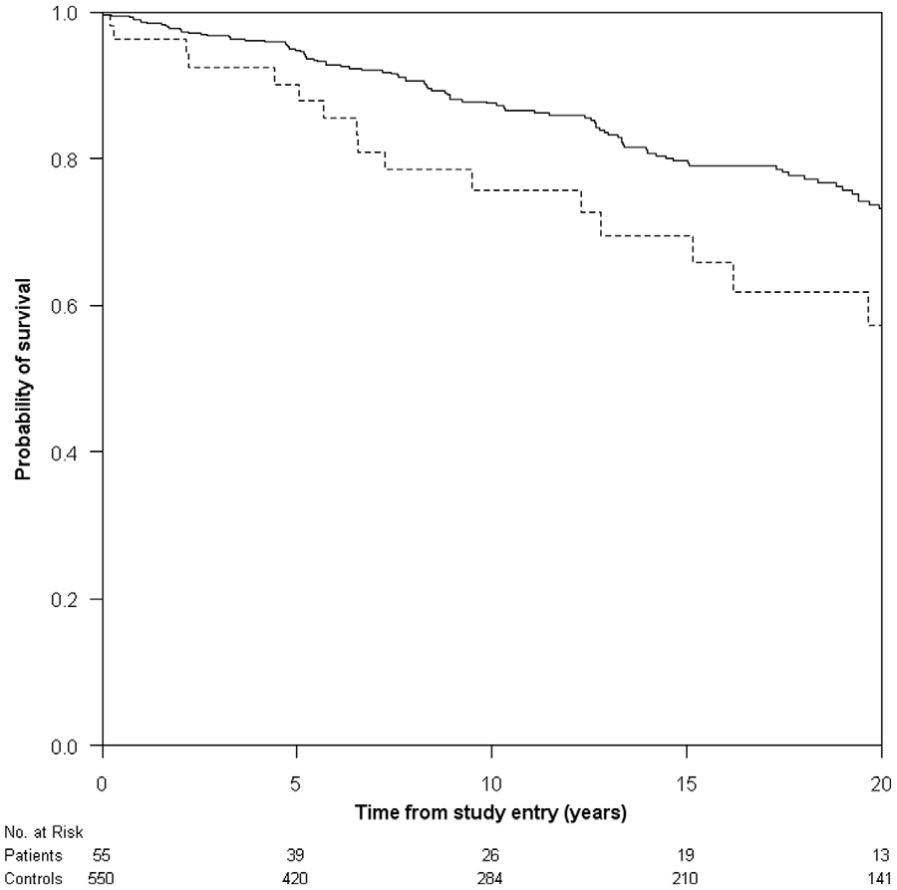
Kaplan-Meier survival curve of patients with tuberculous meningitis (dashed line) and population controls (solid line), Denmark, 1972–2008.

A total of 18 (32.7%) TBM patients and 107 (19.5%) population controls died during the observation period (Table 2). The median age at time of death was 65.1 years (IQR: 54.1–78.9) among the TBM patients and 74.1 years (IQR: 64.0–83.3) in the population control cohort. Figure 1 depicts Kaplan-Meier survival curves for TBM patients and the population controls. The TBM patients were at increased risk of death throughout the entire observation period compared to the population controls. We found an overall MRR of 1.79 (95% CI: 1.09–2.95) for the TBM patients and adjustment for comorbidity did not change the estimate. It appeared, when stratified on individual risk factors, that males were at higher risk of death than women (adjusted MRR 1.99; 95% CI: 0.98–4.07). When stratified on age at time of TBM diagnosis, we found that patients in the 31–60 age group had the highest relative risk of death compared to other age groups (adjusted MRR 2.68, 95% CI: 1.35–5.35). There was a trend towards an increased relative risk of death in immigrant patients (adjusted MRR 2.39; 95% CI: 0.90–6.39).

**Table 2:** Mortality rate ratios (MRR) for tuberculous meningitis patients compared to population controls.

	Unadjusted MRR (95% CI)	Adjusted MRR* (95% CI)	Number of patients who died (%)	Number in the control population who died (%)
All	1.79 (1.09–2.95)	1.76 (1.07–2.91)	18 (32.7)	107 (19.5)
**Stratified on risk factors**				
Sex				
Female gender	1.58 (0.78–3.20)	1.56 (0.77–3.16)	9 (32.1)	57 (20.4)
Male gender	2.05 (1.01–4.16)	1.99 (0.98–4.07)	9 (33.3)	50 (18.5)
Ethnicity				
Native Danes	1.61 (0.90–2.89)	1.60 (0.89–2.87)	13 (52)	86 (34.4)
Immigrants	2.46 (0.93–6.53)	2.39 (0.90–6.39)	5 (16.7)	21 (7)
Age at diagnosis				
16–30 yrs	2.02 (0.24–17.29)	2.01 (0.23–17.19)	1 (7.1)	5 (3.6)
31–60 yrs	2.68 (1.34–5.34)	2.68 (1.35–5.35)	10 (32.3)	43 (13.9)
>61 yrs	1.23 (0.56–2.74)	1.11 (0.47–2.61)	7 (70)	59 (59)

*Adjusted for Charlson Modified Comorbidity Index two years prior to TBM diagnosis.

### Cause-specific mortality

As shown in Table 3, TBM patients were at higher risk of infectious disease related death than the population controls with an adjusted MRR of 42.8 (95% CI: 9.09–201.74). The specific infectious disease causes of death were TB-related in four out of eight TBM patients with infectious disease related cause of death, whereas none of the population controls had a TB-related underlying cause of death. TBM patients were at similar risk of death due to non-infectious disease causes as their population controls.

**Table 3:** Cause specific mortality rate ratios in TBM patients compared with population controls.

Cause of death	Deaths in TBM patients, n	Deaths in population controls, n	Unadjusted MRR* (95% CI)	Adjusted MRR** (95% CI)
Infectious disease related death	8	2	42.70 (9.07–201.11)	42.83 (9.09–201.74)
Tuberculosis of the central nervous system	4	0	-	-
HIV	2	1	-	-
Other infectious diseases	2	1	-	-
Non-infectious disease related death	10	105	1.01 (0.53–1.94)	0.99 (0.52–1.90)

*Unadjusted MRR not calculated for all causes of death due to few events.

**Adjusted for modified Charlson Comorbidity Index score two years prior to TBM diagnosis.

## Discussion

In this nationwide, population based cohort study with up to 30 years of follow-up, we observed a nearly two-fold increased long-term risk of death in TBM patients compared to the background population. The increased effect of TBM on mortality was mainly seen in males; patients 31–60 years of age at time of diagnosis and immigrants. Also, we observed that TBM patients primarily die of infectious disease associated causes, in particular TB-related causes of death. Our study results are of value both to the treating physician and to the patients and their families, who want to know the long-term prognosis after having survived the first year after a TBM diagnosis.

To our knowledge this is the first study to address the issue of long-term mortality in TBM patients in a population-based cohort study design. Previous studies include the initial clinical trials of Streptomycin for the treatment of TBM from the 1940's, where 64% of the patients died within an observation period ranging from four months to just under one year [14]. In a recent case-control study from the United States [15], a hazard ratio for death of 3.72 (95% CI 1.82–7.60) in 135 patients with TBM compared to 75 controls with non-proven TBM with an observation period of maximum nine years was reported. This estimate is higher than our estimate due to the observation period including also the first year after TBM diagnosis. The most frequently reported definitive cause of death was, consistent with our findings, TBM itself. Our study addresses long-term mortality in TBM patients in a more explicit way by comparing mortality in two study groups – one with clearly defined TBM and the other without. Also, our populations are matched on gender and age minimising risk of confounding, and our observation time is significantly longer.

TBM is associated with a high level of morbidity [2], [4], [16], [17]. The most commonly reported neurological sequelae among TBM patients are epilepsy, cognitive impairment, cranial nerve palsy and motor deficits [18]–[20]. Despite these well-documented neurological sequelae, the risk of death from neurology related causes of death was not increased in our TBM patient population. On the contrary, the analysis of cause-specific death revealed that even long-term mortality is registered as TBM being the underlying cause of death.

In a recent large cohort study analysing long-term mortality in Danish patients with meningococcal disease (of which the majority had meningitis) only a slightly increased overall MRR of 1.27 (95% CI 1.12–1.45) was found. Interestingly, these patients were at increased risk of death due to nervous system diseases (MRR 3.57; 95% CI: 1.82–7.00) but not at increased risk of death due to infectious diseases [21]. The excess mortality and increased risk of death due to neurological causes were ascribed long-term sequelae to the disease. Similarly, in a study reporting long-term mortality in patients diagnosed with pneumococcal meningitis in Denmark [22], a high level of morbidity was apparent as patients were at increased risk of death due to nervous system disease but not to infectious diseases in general.

The high MRR reported in our study is noteworthy given that Denmark is a setting of both low TB and HIV prevalence and a country with a well developed national health care system where all patients are treated free of charge. The in-patient mortality rates are generally similar for HIV and non-HIV positive TBM patients [3], [23]. However, when looking at 9-month survival in TBM patients, a Vietnamese study found a significantly increased mortality (MRR 2.91; 95%CI: 2.14–3.96) in HIV positive patients [24].

Even though the main objective of this study was to address the long-term mortality, it is important to underline the observed 32% mortality among TBM patients within the first year of diagnosis. This is higher than the 19% short-term mortality recently reported in a retrospective study regarding TBM patients diagnosed in Denmark from 2002–2008 [4]. This may reflect a trend in declining short term mortality, perhaps due to some progress in diagnostics, such as NAA and magnetic resonance imaging (MRI). Also, dexamethasone has become a part of routine anti-tuberculous meningitis treatment after it proved to significantly reduce short-term mortality rates [25].

A large proportion of the patients in our study had been diagnosed with TB in the year leading to TBM diagnosis. It is unlikely that the subsequent development of TBM could be attributed drug-resistant TB, since drug-resistant TB constitutes a minor problem in Denmark. A recent study showed that 6.1% of all culture-confirmed TB cases in Denmark in the period 2002–2007 had monoresistance or polyresistance to isoniazid [26]. Multi-drug resistant (MDR) TB cases are very rare with a reported incidence of 0.5% from 1992–2007 [27]. The monitoring of drug-resistant TB in Denmark is intensive, as all new cases undergo drug-susceptibility testing at Statens Serum Institute. The general rate of recurrent TB is low in Denmark, as shown in a recent study with a 13.5 year follow-up, where relapse accounted for only 1.3% of the recurrent cases [28]. It is possible that the TBM patients in this cohort had compliance problems leading to an increased risk of relapse; however, this question exceeds the aim of this study.

The apparent effect modification with regards to gender and MRR may be caused by males with TB being more socially marginalized than females with TB. The effect modification with regards to age and MRR is probably a result of an increased mortality in the population control cohort of individuals above the age of 60 years, which leads to a decrease in relative risk of death [29].

There are a few limitations to our study. Because TBM is a rare disease in the Danish population, it was only possible to include a small population of 55 TBM patients reducing the statistical power of our results. We were not able to match our study population on ethnicity; in our stratified MRR calculations, the MRR shows a greater impact of TBM in immigrant patients. However, since the impact of TBM on mortality was greater in younger patients, the increased effect of TBM in immigrants could be explained by the demographic constellation of this group having a greater proportion of younger patients rather than by an effect of ethnicity.

The patients included in this study have all been notified with a TB diagnosis to the DTBR over a more than 30 year period. By pairing data with data on TBM discharge diagnoses from the DNPR we have minimized the risk of misclassifying disease status in the study population. The diagnosis in 76% of the TBM patients was microbiologically verified. There is no risk of loss-to-follow-up bias due to only one study participant falling in this category. The usual risk of confounding attributed to gender and age is not present in our study because population controls were matched on exactly these two factors. The unique and validated Danish registries along with a follow-up time of up to 30 years add further strength to the study.

### Conclusion

In this population based cohort study adult patients diagnosed with TBM were shown to have a life-long increased risk of death compared to the background population. Despite one third of TBM patients dying during the first year after diagnosis, the prognosis remains bleak thereafter – even in a high-income, low TB- and HIV-endemic setting.

## Supporting Information

Appendix S1 **Classification of ICD8 and ICD10 codes used to categorise causes of death.**
(DOC)
