# Supplementary material for: Long-Term Mortality in Patients with Tuberculous Meningitis: A Danish Nationwide Cohort Study
Source: PLoS One. 2011 Nov 22;6(11):e27900. doi: 10.1371/journal.pone.0027900 (PMC3222654; doi:10.1371/journal.pone.0027900)
Supplement: Appendix S1 — Classification of ICD8 and ICD10 codes used to categorise causes of death. (DOC) [file pone.0027900.s001.doc]

**Appendix S1**

The eighteen categories of specific underlying causes of death were specified by ICD Eighth Revision, codes for the years 1977-1993 and ICD Tenth Revision, codes from 1994 to 2006. For infectious diseases, the codes were 000-134.99/A00-B99; cancer 140-209/C00-C96, blood/immune diseases 280-289.99/D50-D89; endocrine diseases 240-279.09/E00-E90; mental diseases/drug abuse 290-315/F00-F99; nervous system diseases 320-358.09/G00-G99; diseases of the sensory organs 360-389.99/H00-H59; cardiovascular diseases 390-458.99/I00-I99; respiratory diseases 460-519.99/J00-J99; digestive system diseases 520-577.99/K00-K93; skin diseases 680-709.99/L00-L99; rheumatological diseases 710-738.09/M00-M99; genitourinary diseases 580-629.99/N00-N99; neonatal/congenital disorders 740-779.99/P00-Q99; pregnancy related diseases 630-678.09/O00-O99; injury/poisoning 800-999/S00-T98,V,W,X,Y; ill-defined causes 780-796.99/R00-R99 and no cause of death reported.
